# Social complexity is not strongly predictive of indiscriminate killing of wartime enemies in a cross-cultural sample

**DOI:** 10.1017/ehs.2025.10033

**Published:** 2025-12-22

**Authors:** Kiran Basava

**Affiliations:** College of Information Science, University of Arizona, Tucson, AZ, USA

**Keywords:** social complexity, warfare, cultural databases

## Abstract

The origins and correlates of war are historically contentious in anthropology, with researchers divided over its relationship to the development of agriculture, sedentism, and centralized states. Although this research tends not to focus on norms of wartime conduct, its arguments can be extended to how the levels and forms of violence directed at enemies vary with social complexity. For this study, variables on social complexity and warfare were coded from ethnographic and historical sources into a cross-cultural data set of 73 societies. The likelihood of different individuals who were enemies of the focal society being targeted or killed during war was tested for relationships with measures of social complexity and violent conquest of external populations. The results of the analyses provided little to no evidence for increased or decreased indiscriminate violence with social complexity (as measured by population size, governance levels, and centralization), or for a strong relationship with formal military structures and political/territorial expansion. A multidisciplinary literature review of how wartime violence relates to social structures is also presented. Interpretations, limitations, and future directions are discussed in the context of comparative cultural databases and their applications to cultural evolution research.

## Graphical abstract

**Figure:**
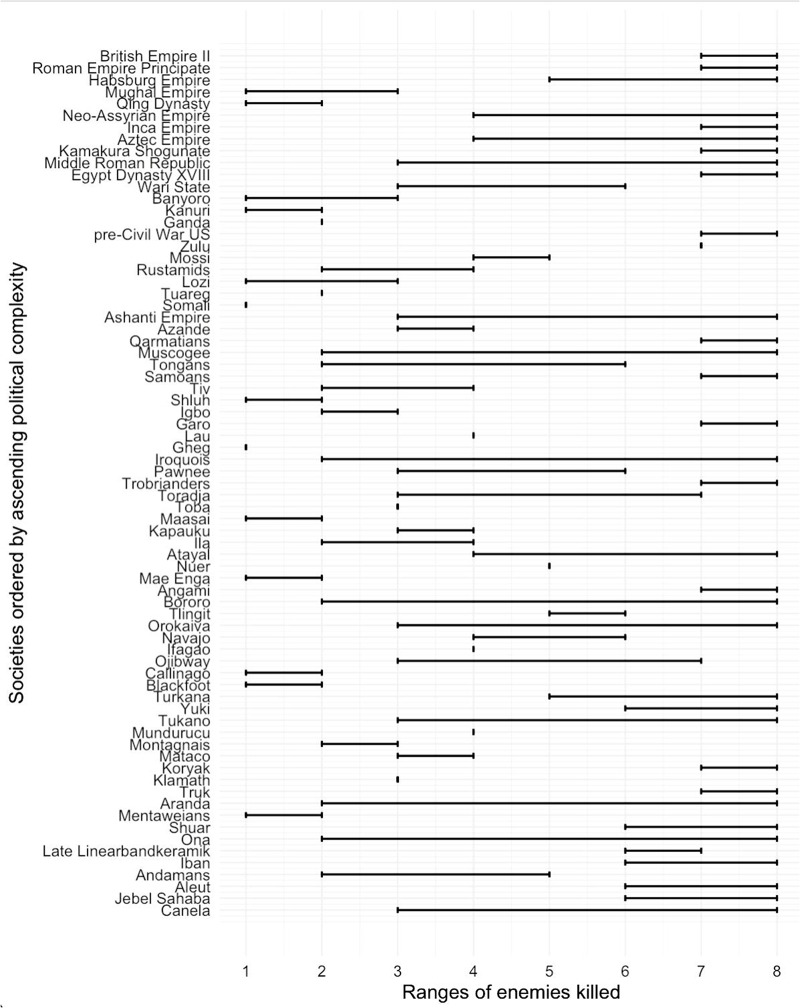

## Social media summary

Social complexity is not strongly predictive of indiscriminate killing of wartime enemies in a cross-cultural sample

## 1. Introduction

An overall trend in human history is increased scales of cooperation in larger and more complex societies. However, there is no scholarly consensus on how this has affected the intensity and forms of violence in war, including how enemies are treated. In other words, it is unclear whether and how the idea of an expanding moral circle, that is, considering more distantly related or unrelated people (and possibly other living organisms) to be worthy of some level of care and moral treatment (Singer, 2011), can be applied to the conduct of war. There is an extensive literature debating the extent of warfare among human societies pre- or post-farming or sedentary living, and how the intensity of war has varied in relation to predominant types of sociopolitical structures from early human groups to the current day (e.g. Fry, 2013; Haas, 2001; Keeley, 1996; Kelly, 2000; Kim & Kissel, 2018). Archaeological, ethnographic, and historical evidence indicates how warfare tactics may range from raids conducted by nomadic bands, battles and massacres between chiefdoms, months- or years-long sieges between state armies, and other forms in between (Kim et al.,^2015^), each with different consequences for the levels of violence directed at peoples on both sides, including those not directly involved in combat (Hatch, 2017; Otterbein, 2000). This study investigates whether a specific type of wartime violence – the indiscriminate killing of enemies – varies with measures of social complexity in a cross-cultural data set. ]

War has been defined differently by various researchers, but for the purposes of this study, it is defined as organized lethal violence between social groups (for other definitions and debates, recent reviews include Glowacki et al., 2020; Hames, 2019; Kissel & Kim, 2019). Importantly, this current definition does not distinguish between internal and external war: if a group of people were engaged in collective violence against another group, for the purposes of this study, that would qualify as violence against an enemy. Social distance is an important factor for severity of war (Solometo, 2008), but as identities of enemies were not always clearly described in the sources, this was not explicitly considered in the current analysis.

Social complexity here is used as a non-normative term consistent with its meaning in the anthropological and archaeological literature, which encompasses factors such as population, territory, and settlement sizes, levels and degree of specialization of governmental institutions, and centralization of political authority, among others (Turchin et al., 2018). Evidence from multiple regions indicates that these factors may appear in different orders, degrees, and temporal proximities (Feinman, 2013). However, there are still cross-cultural commonalities in the history of different regions, including the existence of broadly comparable forms of sociopolitical organization which range from small-scale, politically decentralized groups to large empires, with an overall trend of greater social complexity in most regions over time (Currie & Mace, 2011; Haas, 2001; Turchin et al., 2018).

### 1.1 Patterns of war and social complexity

There has been extensive debate around the extent to which war was present among small-scale foraging societies thought to be representative of early human history (Ember & Ember, 2014; Glowacki, 2024; Hames, 2019; Otterbein, 1999). Some of this disagreement can be attributed to differing definitions of war, difficulty with assessing effects of pacification in the ethnographic record, and uncertainty about the representativeness of archaeological evidence for war in the Pleistocene (Ember & Ember, 1992, 2014; Ferguson & Whitehead, 1992; Hames, 2019; Kissel & Kim, 2019). Overall, extensive surveys of ethnographic literature as well as archaeological evidence have indicated that war – as defined above – does occur among mobile foragers and other small-scale, non-state societies, although it varies in scale, frequency, and severity (Ember & Ember, 2014; Kim & Kissel, 2018).

Although this research tends to focus on frequency, causes, and mortality, it can point to certain patterns of violence and consequences for enemies in nonstate war. Broadly, such societies do not have the formal military organizations that come with centralized political authority. Instead, they rely on widespread voluntary participation in fighting by adolescent and adult males. War is conducted through raids and ambushes on living spaces of other groups with little separation between combatants and non-combatants (Keeley, 1996). Such tactics could facilitate greater levels of indiscriminate violence and in some cases extermination of enemy groups (Keeley, 1996; LeBlanc & Register, 2003). These patterns are corroborated by archaeological sites with skeletal remains, particularly Jebel Sahaba (∼13,000–18,000 years ago), which provides evidence for repeated intergroup violence encompassing male and female children and adults (Crevecoeur et al., 2021), and Nataruk (∼10,000 years ago), purported to show the massacre of a foraging community (Mirazón Lahr et al., 2016). However, it is unclear how representative sites like Jebel Sahaba and Nataruk are of small-scale warfare in the late Pleistocene/early Holocene. Raids and ambushes may not have left visible archaeological traces such as the fortifications and specialized weapons apparent from later settled societies (Kissel & Kim, 2019). Alternatively, these sites could represent exceptional events. There is clearer evidence for intensified war with early farming societies corresponding with settlement consolidation and population growth in multiple regions (Arkush & Tung, 2013; Haas, 2001; Kim et al., 2015; Underhill, 2008). Mass graves are present at several sites in Neolithic Europe with the development of agricultural lifeways (Fibiger et al., 2023; Meyer et al., 2018). While the presence of all ages and sexes at some sites illustrates indiscriminate violence, other sites have demographic variation that may indicate captive-taking of women and children (Meyer et al., 2018). Factors implicated in such studies include resource competition in conjunction with increased inequality and social stratification (Fibiger et al., 2023), possibly exacerbated by climatic disturbances (Gronenborn, 2006).

With the development of more centralized political structures, coercive processes of expansion may have led to intensified violence on frontiers, particularly with technological advancement and military specialization (Kim et al., 2015; Otterbein, 1970). Based on a cross-cultural sample of 42 societies, Otterbein (2000) concludes that centralized societies were more likely to kill women and children than were uncentralized societies, and that despotic early states, chiefdoms, and ‘dependent native peoples’ were most likely to kill captives and non-combatants. He writes that these findings contradict those of an earlier study by Hobhouse et al. (1915), which found decreased killing of captives with higher agricultural stages. In contrast, his findings are somewhat consistent with Ember et al.’s (2013) findings in eastern Africa that state societies were more likely to commit atrocities during internal war. However, Ember et al.’s results indicated states were not significantly more likely to kill combatants and non-combatants in external war (Ember et al., 2013). Both works note how the social meanings of wartime violence and the resulting treatment of enemies changed with increased political consolidation among state societies. This is consistent with analyses of the integration of military and political authority and greater social importance of militaristic norms in early states (Arkush & Tung, 2013; Haas, 2001; Kim et al., 2015). In particular, Ember et al. write that a connection between ‘military glory’ and behaviours like killing non-combatants, torture, and trophy-taking in war exists in state but not non-state societies (2013, p. 51). Otterbein (2000) similarly links the killing of captives and non-combatants, as well as the use of torture and mutilation, with strategies of terror used by hierarchical ‘despotic states’ to consolidate power. It is possible that reduced interdependence and increased social distance from enemies in state societies could facilitate killing non-combatants (Ember et al., 2013), although this is complicated by other incentives – taking over territory for land could have more exterminatory results than taking it for political conquest and/or slaves (Otterbein, 2000). Therefore, specific material and political objectives of war could determine the expedient treatment of people residing in a territory desired for conquest by an invading polity.

Ember et al. (2013) note that their results of greater atrocities in state societies may in part be due to the tendency of ethnographically recorded state societies to be autocratic and dependent on violent coercion, in line with Otterbein’s description of despotic states. It is possible that among less autocratic states with professional military structures, increased social and physical distance between combatants and non-combatants might decrease exposure of populations to external violence. This contrasts with what is effectively total war among small-scale societies (Keeley, 1996). There may also be pacification of conquered areas through increased political control and centralization (Glowacki, 2024; Snyder & Arkush, 2024). These arguments are combined with claims about shifts in moral norms by Pinker (2011). He argues for a decline of warfare (and other forms of violence) first during the development of agricultural societies and later in seventeenth- and eighteenth-century Europe, emphasizing the role of organized states in reducing the severity of war and in promoting greater reason and empathy. However, there have been extensive critiques of Pinker’s data and conclusions: researchers have questioned the criteria used to group together deaths occurring across widely varying amounts of space and time to illustrate how deaths from violent conflict have supposedly declined (Falk & Hildebolt, 2017; Kim, 2012; Mann, 2018). His estimates for deaths in historical warfare have been described by relevant experts as highly exaggerated with unclear or inappropriate sources (Butler, 2018; Dwyer & Micale, 2021; Ferguson, 2013; Fibiger, 2018). As noted above, there is archaeological evidence for increased intergroup violence among early farming societies in comparison with foragers (Fibiger et al., 2023). With regards to the formation of modern organized states, ideologies and institutions regulating internal violence could occur alongside extreme external violence during colonial projects (Kim, 2012; Mann, 2018). Two studies (Falk & Hildebolt, 2017; Oka et al., 2017) apply scaling relationships to estimates of conflict deaths in relation to population size. Falk and Hildebolt (2017) find that war deaths scale with population size regardless of social organization and conclude that ‘people living in small-scale societies are not inherently more violent that those living in “civilized” states’. Oka et al. (2017) model war group size and population size and find that lethality is not higher among small-scale than state societies, whereas among large societies efforts to eliminate enemy populations may result in higher levels of non-combatant deaths. Although mortality estimates are a valuable measure of the scale and severity of war, they do not necessarily track moral attitudes about the limits of wartime violence in the participating societies. Below, more qualitative work on social norms and potential drivers of indiscriminate violence is reviewed.

### 1.2 Norms of restraint

There is historical evidence for mutually understood norms of conduct between centralized small polities which frequently fought one another in multiple regions, such as in pre-colonial western Africa (Smith, 1989) and in early Greek and Roman warfare (Raymond, 2010). These codes of conduct may have become more common and detailed with the formation of complex polities, and often centred around views of a natural or divinely ordained state of relations between human groups. However, these did not necessarily provide any protection for non-combatants or limits on other forms of violence. Instead, rules of conduct during war were often used to defend the divine order of rulers and their territories from chaotic outsiders (Brekke, 2005; Cox, 2017). For instance, the concept of *ma’at* in ancient Egyptian war characterized foreigners as barbarians rebelling against the order and justice of the pharaoh. This legitimized offensive war, including widespread captive-taking and slaughter of defeated enemies, as self-defence and law enforcement (Cox, 2017). While the reasons and means of waging war may have taken place within a system of moral thought centred on order, civilization, and divine rule, this appears not to have resulted in consideration of the morality of how enemies were treated. Some scholars have argued for a decrease in these extreme displays of violence with the development of moralizing, broadly prosocial ideologies with major religious traditions (Bellah, 2011). In particular, there are similarities in institutions and aspects of moral thought across many of these traditions, including concepts of just war (Abou El Fadl, 2001; Brekke, 2005; Johnson, 1981).

While less studied in this context, small-scale societies often had parallel forms of restraint. These include protection for some classes of people based on their supposed incapacity to fight (women, children, the elderly or disabled) or social occupation/class marking them as peaceful (religious officials, diplomats, peasants). Among the Mae Enga of Papua New Guinea, norms included immunity for women and children, the wounded, refugees, or people from a clan with whom one was not at war (Wiessner, 2019). Similar limits to violence in war can be found across many societies with different emphases and inclusions/exclusions of particular individuals or actions (Chirot & McCauley, 2010; Hasluck, 1954; Straight, 2017). Other common restrictions included forbidding the use of certain types of weapons, killing wounded enemies or captives, or bodily mutilation (Keeley, 1996). Norms based on social distance were also common and delineated applications of the other restraints (Solometo, 2008). For instance, cultural familiarity was central to Mae Enga codes, which developed as part of an interclan exchange and reparations system that maintained a balance of power and provided an avenue for status-seeking through constrained war and peacemaking (Wiessner, 2019). Such codes could of course be highly variable. Many societies were recorded as regularly killing men, women, and young children without much distinction unless they were taken as captives or slaves (Straight, 2017). Beliefs about proper conduct could also vary within societies, as illustrated by a recent study on Turkana pastoralists that found high individual variation in beliefs about the acceptability of killing individuals of different ages or genders (Zefferman & Mathew, 2020). Unfortunately, as Keeley notes, ‘ethnographers have seldom asked individuals – men or women – about their attitudes toward and reactions to war’ (Keeley, 1996, p. 146), so the justificatory mechanisms surrounding these three types of combat can in most cases only be inferred indirectly from the actions themselves and records of the cultural systems surrounding them.

### 1.3 Mass killing and civilian victimization

Research on mass killing and deliberate targeting of civilians offers an additional perspective on social complexity, in the form of state capacity, ideology, and resultant forms of violence. This work indicates that exclusionary ideologies, through which societies premise their identity on excluding unwanted groups from their circle of moral consideration, are a consistent contributing factor in genocide (Bellamy, 2012; Harff, 2003; Nyseth Brehm, 2016). This can be manifested through a group’s belief in its higher status and responsibility to civilize others. For instance, norms developed in Europe for protecting civilians, eventually codified in the Geneva conventions, were generally understood as not applying to the ‘uncivilized’ peoples living in colonized areas (Bellamy, 2012). A common theme in extermination attempts is a desire to return to an idealized past utopia of racial purity, often tied to a romanticized view of pastoral life and a right to conquer and cultivate lands wasted on their current-day, non-farming inhabitants (Kiernan, 2007). These exterminatory worldviews are also consistent with arguments for greater externalization of violence among complex societies engaged in conquest or colonization (Kim, 2012). It is therefore difficult to claim that violence in war decreased with the increase in codified restraints in state societies of any formulation. The present-day need to justify rather than glorify mass slaughter may document a shift in norms of acceptable behaviour in a globally interconnected human community more than actual changes in violence.

Another focus of this research is how extreme violence may be used instrumentally to achieve political goals; namely, when actors view mass killing as less costly than any alternatives (Chirot & McCauley, 2010; Straus, 2012). In particular, insecure power structures may use mass violence in response to perceived existential external or internal threats (Downes, 2006; Straus, 2012; Valentino, 2014). This reasoning can apply in different senses to both ancient polities as described above and contemporary nation-states. It is also consistent with the use of terrorism by relatively weak insurgent groups as part of an overall pattern of asymmetric war (Valentino, 2014). These descriptions indicate that levels of violence in war would not necessarily track levels of social complexity (either positively or negatively) but be more pronounced among somewhat centralized political systems lacking security and stability.

Centralized states have greater organizational ability and physical resources to carry out extermination campaigns. However, this capacity for destruction is in absolute rather than proportional terms and does not necessarily indicate more exclusionary morality on the part of states. (Likewise, greater destruction in proportional terms among nonstates does not necessarily indicate the reverse.) Based on this literature, a possible conclusion is that mass killing as an end in itself applies more to social groups with political goals beyond resource acquisition or revenge. This contrasts with the collateral, incidental forms of indiscriminate killing often described in the ethnographic literature on small-scale societies. There are instances of exterminatory warfare among small-scale societies as well (Burch, 2005; Karsten, 1935; Keeley, 1996). However, driving factors such as fear of pollution by ideologically or ethnically impure groups, perceived threats to a centralized regime, or desire to achieve a utopia are less likely to be present among these groups. Instead, the motives for war described as exterminatory in intent cited in ethnographies include preventing the enemy from returning to enact revenge (Jivaros, Karsten, 1935), taking possession of the defeated’s territory (Chuuk, Fischer, 1958), and fear of neighbouring population growth impinging on a society’s land and resources (Crocker & William, 1990). Based on this data, motives for exterminatory war resemble those for warfare overall among small-scale societies, centring around revenge and resource acquisition.

Multiple factors may promote or restrain indiscriminate violence, including a society’s past experiences of conflict, its internal social structure, norms, and ideologies, and perceptions of threat from other groups or the environment (Chirot & McCauley, 2010; Glowacki et al., 2020). Even if the evidence ultimately supports one position or another regarding the historical trajectory of war, attitudes towards outgroup members, norms of combat, and levels and discrimination in the use of violence may not necessarily correlate with the scale, frequency, or effectiveness of war as it has coevolved with social complexity over history. It is therefore possible that no linear relationship will appear between measures of social complexity and indiscriminate killing in war due to overwhelming variation across cultures (or, more pessimistically, a consistently high level of indiscriminate killing).

## 2 Hypotheses

**Figure 1.**
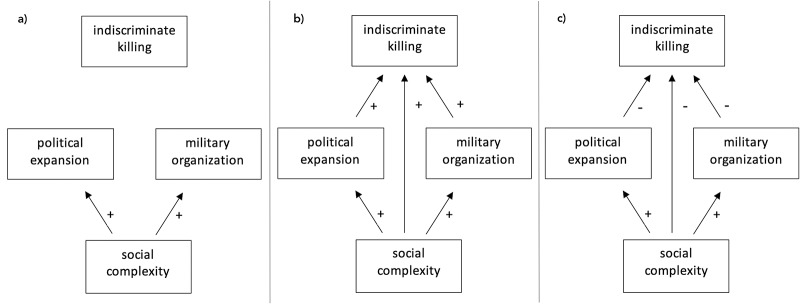
Diagram representing (a) the null hypothesis, (b) the first alternative hypothesis, and (c) the second alternative hypothesis on the posited relationships between social complexity, aims of war, form of military organization, and indiscriminate killing.

This paper uses a global data set of societies coded from archaeological, ethnographic, and historical sources to test whether the likelihood of different individuals who were enemies of a focal society being targeted/killed during war is correlated with measures of social complexity and violent conquest of external populations. The following hypotheses were tested (illustrated in Figure 1):

*Null hypothesis. There is no clear relationship between measures of social complexity and levels of indiscriminate killing during war.*

Rationale: No linear or consistent relationship exists, due to the numerous factors separate from or inconsistently related with social complexity that also affect levels of violence and restraint during war.

*Alternative hypothesis 1. Measures of sociopolitical complexity are positively correlated with indiscriminate killing of enemies during war.*

Rationale: Despite increased separation between fighting forces and the rest of the population, more indiscriminate killing occurs with increased social complexity due to an increased scale of warfare with political expansion and more effective military infrastructure.

*Alternative hypothesis 2. Measures of sociopolitical complexity are negatively correlated with indiscriminate killing of enemies during war.*

Rationale: In contrast with larger complex societies with formal militaries, reliance on surprise attacks and a lack of distinction between combatants and non-combatants among small-scale societies will result in more indiscriminate killing.

## 3 Materials and methods

### 3.1 Data set construction and sampling

**Figure 2.**
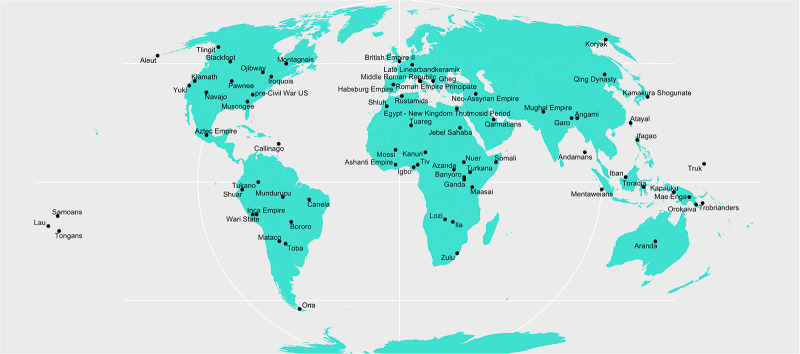
Geographic distribution of the societies in the data set.

For this study, data were coded from ethnographic documents accessible in the eHRAF World Cultures database, other ethnographic sources including the Ethnographic Atlas (Murdock et al. 2025) and Standard Cross-Cultural Sample (Murdock & White, 2025) accessed through D-Place (Kirby et al., 2016), and secondary historical sources, as well as data stored in the Seshat Global History Databank (Turchin et al., 2015). The final sample consists of 73 societies documented as cultural units in these sources. Although this is clearly a tiny fraction of the breadth of human history and culture, an attempt was made to create a sample somewhat representative of different geographic regions and levels of social complexity for societies that engaged in war and for which sufficient descriptions of treatment of enemies were found in the literature. However, it should be noted that this is not a probabilistic sample such as the eHRAF Probability Sample Files (PSF; Naroll, 1967), as the main objective was to find relevant descriptions of war in the available materials, so generalizability may be limited. For instance, there may have been biases around which societies had ethnographic records describing the conduct of war. A world map showing the geographic distribution of societies is shown in Figure 2.

To construct the data set, eHRAF subject categories, particularly those on armed forces and war, as well as keywords were used in initial searches to find societies for which there was some description of warfare. Materials in eHRAF have been indexed according to the Outline of Cultural Materials (OCM), which contains a range of broad subjects ranging from religion, family life, subsistence, and other aspects of human societies (Ember & Ember, 2013). Specifying relevant subject categories when searching eHRAF is an established method for finding relevant ethnographic records to compile data sets for cross-cultural studies (e.g. Ember et al., 2013). For the purposes of this study, the OCM categories 700 (armed forces) and 720 (war) were primarily used. Keywords potentially contained in relevant descriptions may also be used to narrow eHRAF search results (Fischer & Ember, 2018). For this study, keywords related to violence against enemies in war were used to search across and/or within subject categories. A full list of the keywords is in the Supplementary Material. The keywords used would sometimes be adjusted based on the amount of material on warfare for different societies as well as the content of that material: for societies with extensive descriptions of warfare, fewer, more direct keywords were used whereas societies with limited descriptions of warfare were searched with additional, broader keywords to find any relevant descriptions. Those descriptions were then used to code the target variable of indiscriminate killing.

To expand the data set to include historical and archaeological societies, Seshat polities that had been coded with some degree of certainty (present/inferred present or absent/inferred absent) for variables of interest in the ‘Warfare Intensity’ section (e.g. general massacre and extermination) as of 2020 were also added. Although different variables on wartime behaviour were created for this study, these Seshat variables provided an indication of available data and sources on indiscriminate killing. The sources and evidence for the codes on massacre/extermination were checked for each polity included; those for which the evidence was found to be insufficient and no additional sources were found were removed. Societies which could not ultimately be coded for the outcome variable of indiscriminate killing due to insufficient descriptions in the primary/secondary sources were removed. Overall, 54 societies of 73 total were primarily coded from ethnographic sources, generally accessed through eHRAF, 13 from secondary historical sources, and 6 from papers with archaeological data. Fifty-nine societies have a start date after 1500 bce, and 41 of these have a start date after 1900 ce. As ethnographies tended to provide greatest detail for such treatment and these were carried out in more recent times, a majority of the sample, 56 societies, have an end date after 1900 ce.

It should also be noted that this study does not include data for nation-state conflicts from World War I onwards (societies dated after this period are non-state, ethnographically recorded societies). Extensive quantitative work on genocides and civilian targeting in the context of modern nation-state war has been carried out in political science and genocide studies (e.g. Downes, 2006; Eck & Hultman, 2007; Harff, 2003). Instead, this study attempts to take a less detailed but broader historical perspective on the relationship between wartime violence and social complexity across different cultures.

### 3.2 Variables

The predictor variables were social complexity as measured through a principal component analysis of population estimates, degree of centralization, and hierarchical governance levels as coded in the above databases for each society; presence of a formal military structure, and violent political conquest/expansion (full descriptions of the variables in Supplementary Material).

The outcome variable of enemies killed in external war was defined as whether enemy individuals in the following age/gender categories tended to be killed during war: male or female infants/toddlers; children; younger adults; older adults. One point was added for each age/gender category with the minimum being 1 and the maximum being 8 (everyone). Ranges were used for some societies when there was ambiguity or overlap across categories. An overview of these codes is shown in Table 1.

**Table 1.** Possible codes for enemies killed in war

Killed in war	Female	Male
Infants/toddlers	0−1	0−1
Children	0−1	0−1
Younger adults	0−1	0−1
Older adults	0−1	0−1
Total	0−4	0−4
Hypothetical minimum = 1		
Hypothetical maximum = 8		

For instance, the following description of the Aleut was used to code them as 6–8 (all killed except for some who are taken as wives or slaves): ‘The Aleut was a ruthless warrior. He attacked other villages for revenge, new wives, rare stones for charms and weapons, and perhaps most significantly, to divert aggressive impulses to outsiders. War parties, usually led by a chief or one of his relatives, conducted surprise attacks on other villages (Aleut and Eskimo), frequently murdering every person in the village. Prisoners brought back to the villages were accorded diverse treatments. Some became wives and were integrated into the community. Others remained in slave status to be used or abused according to the whim of the owner’ (Jones, 1970, p. 35). Codes and descriptions for each society are in the supplementary file ‘inkill_dataevidence.xlsx’.

Age and gender are not necessarily the most salient reasons for why enemies would be killed or spared in a conflict, but scoring in this manner ideally captures variation in scales of violence less arbitrarily than a more undescriptive categorical or numerical scale. There was a lack of evidence for any norms constraining the extent of violence or protection for enemy individuals in most of the societies surveyed. However, it cannot be assumed that for all societies where no limits of war are found in the sources that indiscriminate violence was normal or desirable – the presence or absence of such evidence may result from the vagaries of historical records or the interests of specific researchers. With these caveats, this variable is intended as a proxy for the regular and presumably accepted means of conducting war in these societies. Generally, societies coded 1–3 are those that tended to target adolescent to adult males participating in combat, while those with higher scores, at least based on available descriptions, tended to direct violence more broadly against enemy populations.

### 3.3 Processing of data pre-analysis

Political centralization, hierarchical levels, and population were treated as continuous variables; military organization and political expansion were treated as binary. The first factor from a principal components analysis (PCA) (using the prcomp() function, *stats* package in R) on the population, centralization, and hierarchical levels variables was used as a measure of social complexity (cumulative proportion of variance explained = 0.88). Political centralization, hierarchical levels, and population were standardized (mean centred at 0 with a standard deviation of 1) prior to the PCA. Missing data were imputed using the R package *mice* (van Buuren & Groothuis-Oudshoorn, 2011) using the methods provided for continuous data (predictive mean matching). This was a total of seven datapoints prior to conducting the PCA: five societies missing data for population and two for hierarchical complexity. To ensure basic assumptions of the hypotheses were met, logistic regression models on political expansion and military organization as predicted by the social complexity measure (henceforth PC1) were run. Both variables were predicted by PC1 (log odds = 1.51, *p* < 0.001 and log odds = 1.40, *p* < 0.001 for expansion and military, respectively).

### 3.4 Statistical models

**Figure 3.**
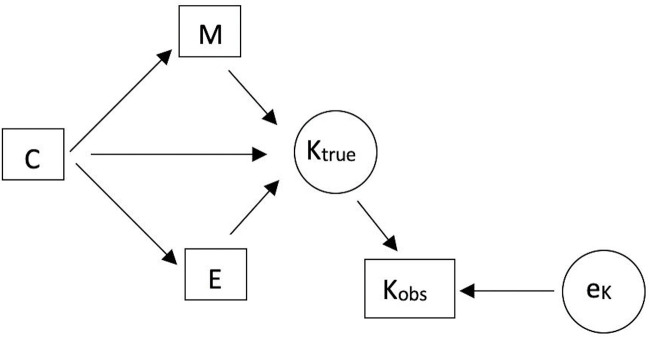
Directed acyclic graph showing hypothesized relationships between the described variables, with measurement error on the outcome. C = social complexity, E = political expansion, M = military organization, K_true_ = enemies killed (true value), K_obs_ = enemies killed (observed value), e_k_ = measurement error for observed killing.

Analyses were conducted using the *brms* package (Bürkner, 2017). A set of multiple regression models were used to test effects of military organization, social complexity, and expansion on the outcome variable. They were designed to test the relationships implied by the graph in Fig. 3. The models were constructed based on the adjustmentSets() function in the *dagitty* package (Textor et al., 2017) that, based on the provided graph, returns the necessary covariates for which to adjust when estimating an effect between two variables. For this graph, the following covariates were returned (for predicting the total effect of the given exposure): social complexity when killing is predicted from military organization; social complexity when killing is predicted from expansion; and none when killing is predicted by complexity. Another model was also run with all three predictors to assess effects of complexity while accounting for the other two variables and to check consistency of estimates.

Models were specified as follows, with one model with all three predictors and then dropping one term each. Priors were chosen to be weakly informative (Prior Choice Recommendations n.d.). To incorporate uncertainty in the outcome variable of enemies killed, it was modelled as an unobserved true value distributed normally with a mean of the observed value (equal to the mean of the range for each society) and standard deviation calculated from the difference from the maximums/minimums and means (based on the model incorporating measurement error in McElreath, 2020, chapter 15 as implemented in *brms* by Kurz, 2019):

K_obs_ ∼ Normal(K_true_, K_se_) *[distribution for observed values of enemies killed]*

K_true_ ∼ Normal(µ, σ) *[distribution of unobserved true values]*

µ = α + β_C*C + β_E*E + β_M*M *[linear model with complexity, expansion, and military as predictors]*

α ∼ Normal(4, 1.3) *[intercept prior]*

β ∼ Normal(0, 1) *[slopes prior for complexity, expansion, military]*

σ ∼ Exponential(1)

Given the ways in which the outcome variable might vary depending on context, it seems unlikely to be influenced significantly by cultural phylogeny or spatial proximity (perhaps excluding cases where two societies fought one another). However, a simplified model using the score ‘kill_mean’ without the error term was run using world region as a varying intercept and compared to a model without it. As there was also a possibility of bias due to the large number of societies in the data set dated to the nineteenth and twentieth centuries when much ethnographic fieldwork was conducted, another model was run with all three main predictors as well as the mean date (standardized) for each society as another predictor.

### 3.5 Preregistration and data availability

The analyses were preregistered prior to conducting the main analyses (after the PCA and imputation) on the Open Science Framework site (https://osf.io/nskct). The data set with sources and code for analyses are available at https://github.com/kcbasava/historical-war-practices/tree/main/indiscriminate-killing.

## 4 Results

**Figure 4.**
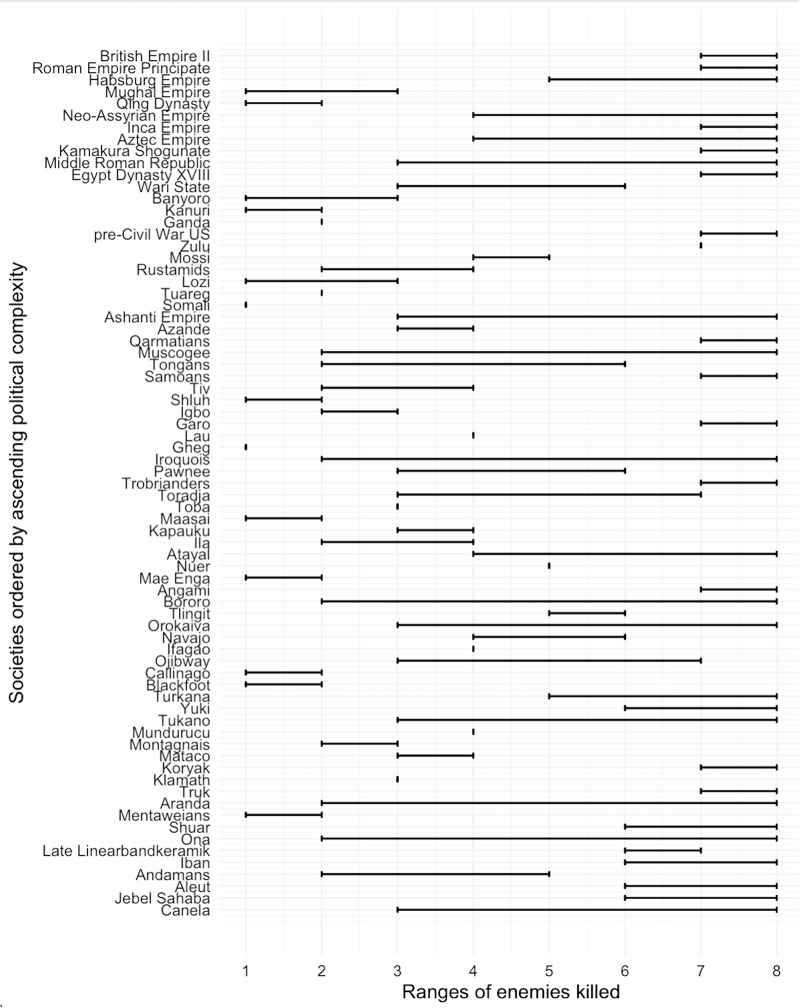
Distribution of enemies killed plotted by PC1 (*y*-axis, ascending from least to highest complexity). Lines are ranges for each society on the 1–8 scale (*x*-axis).

There was no strong evidence for relationships in either direction between the scale of enemy individuals targeted and measures of social complexity, military organization, or political expansion. Overall, 33 societies had the score of 8 for the ‘kill_max’ variable, indicating that on at least some occasions violence would be indiscriminate with regard to sex or age. Two societies had a maximum score of 1, indicating norms limiting violence to only one age/sex category (presumably young men). Thirteen societies had a minimum score of 1, indicating that on at least some occasions only male warriors were killed. The distribution of ranges for enemies killed across levels of social complexity is shown in Fig. 4.

The estimates for the effects of social complexity were centred around 0 (−0.13 in the full model, 95% CI = [−0.83, 0.58]). The estimates for military formalization were slightly negative across models (−0.59 in the full model, 95% CI = [−1.73, 0.54]). The estimates for effects of political expansion were positive across models (1.04 in the full model, 95% CI = [−0.21, 2.20]). However, in all cases the 95% CI included zero. Model coefficients are shown in Table 2. Model comparisons are shown in Table 3. When adding region as a varying intercept in the simplified model with kill_mean as the outcome, the estimates were nearly identical (95% CI = [−0.42, 0.48] when including region as a varying intercept; 95% CI = [−0.41, 0.46] for the model without it) and included 0 in both cases. The model incorporating mean date for each society as a predictor also did not produce noticeably different estimates (full results in Supplementary Tables S1–2). Therefore, it does not appear that the current data provide evidence for an effect of social complexity on indiscriminate killing, either on its own or through military organization, although there is a possible effect from political/territorial expansion. Overall, it appears that the null hypothesis of no clear relationship between these variables is best supported by the analyses.

**Table 2.** Coefficient table for the four main models, listed from left to right in order of model fit as evaluated by leave-one-out cross-validation with the model_weights() function in brms. From left to right, estimates and 95% CIs for m_pem (predicted by complexity, controlling for effects through expansion and military), m_ep (expansion, controlling for complexity), m_p (complexity only), and m_pm (military, controlling for complexity)

	All predictors		Complexity + expansion (estimates)		Complexity		Complexity + military	
Predictors	Estimates	CI (95%)	Estimates	CI (95%)	Estimates	CI (95%)	Estimates	CI (95%)
Intercept	4.47	3.65, 5.32	4.24	3.53, 4.97	4.64	4.12, 5.16	4.83	4.12, 5.56
Complexity	−0.13	−0.83, − 0.58	−0.3	−0.95, 0.36	0.03	−0.48, 0.52	0.17	−0.46, 0.79
Expansion	1.04	−0.21, 2.20	0.94	−0.30, 2.10				
Military	−0.59	−1.73, 0.54					−0.43	−1.58, 0.69
Observations	73		73		73		73	
*R*^2^ (Bayes)	0.052		0.035		0.006		0.02

**Table 3.** Models compared using leave-one-out (LOO) cross-validation with the loo() function in brms. First column is difference in ELPD (expected log predictive density) from the best fitting model and second column is ELPD for each model

	Included predictors	ELPD_diff_	ELPD
m_pem	All (complexity, expansion, military)	0	−162.079
m_ep	Complexity and expansion	−0.17248	−162.252
m_intercept	None (intercept only)	−0.25917	−162.338
m_p	Complexity	−1.37025	−163.449
m_pm	Complexity and military	−1.45943	−163.539

## 5. Discussion

Based on these analyses, the null hypothesis of no consistent relationship between the degree of indiscriminate killing and aspects of social complexity is best supported. It could be argued that the positive and negative estimates for expansion and military formalization, respectively, indicate that societies that engaged in territorial and/or population expansion had somewhat higher levels of indiscriminate killing, while those with military formalization had lower levels. In particular, although the confidence intervals in both cases always included 0 across models, the estimates for expansion were positive across models. This may be due to greater violence against resistant populations by expanding polities, as discussed in archaeological literature on early states and shown in work including Otterbein’s (2000) cross-cultural analysis of killing of captured enemies.

However, given the limitations of the sample and the degree of uncertainty in the results, the null hypothesis can be viewed as the best supported. As reasons for both decreased and heightened amounts of violence across different levels of social complexity were discussed earlier, potential issues with the methods are highlighted here. In particular, some limitations with using quantitative methods to answer the current research questions are apparent with the definition of the outcome variable. While it was intended to capture the somewhat subjective concept of what would be considered the normal and expected targeting and killing of enemies for a given society, it does not incorporate attitudes or intentions behind what might be similar levels of wartime violence. For instance, some societies had restraints on violence against certain classes of enemy individuals, either in practice or in religious/moral codes. These were often linked to the levels of agency attributed to different types of people in these societies and the perceived relevance of that agency. For instance, although Islamic scholars from major schools generally stipulated that violence against women, children, the elderly, and other individuals thought lacking ‘the general competence to fight’ should be avoided, it became permissible in the event these individuals took up arms or engaged in other actions equivalent to fighting (Syed, 2013, p. 148). In contrast, ethnographies of the Kapauku Papuan people during the twentieth century describe how very small male children in enemy villages could be killed despite posing no physical threat, whereas warriors were forbidden to injure women and girls even if they were successfully attacking the men (Pospisil, 1958). This distinction between protection based on actions versus identity is one instance of variation between societies treated equivalently in the coding system used, and which also would not have a clear relationship with degree of social complexity. Additionally, the outcome variable does not distinguish between truly indiscriminate violence without specific targets or intentional destruction of particular group as an end in itself, a distinction which has been pointed out by political scientists studying genocide and patterns of violence in contemporary war (Straus, 2012).

Using individual conflicts or violent events as units of analysis, which is the approach taken by many databases of contemporary conflict (e.g. Eck & Hultman, 2007), was not done here because one of the aims was to determine the possibility of observing consistent within-society and distinct between-society patterns of behaviour in war – in this respect, characterizing the average behaviour of a society in war is different from sampling individual conflicts. However, this may have further obscured variation within cultures. In particular, the codes for some societies covered long time periods during which norms of combat likely changed. This was partly mitigated by attempting, as much as possible, to ensure the evidence for indiscriminate killing referred to the same time period as evidence for the social complexity variables. However, there is still potential mismatch in the codes for social complexity variables and codes for indiscriminate killing. A possible future direction could be to examine available data on a society’s individual conflicts to characterize both between- and within-societal variation. Additionally, although the current results indicate no difference in levels of indiscriminate killing between societies with and without professional military structures, this variable is an oversimplification of a variety of ways in which military forces can be organized and mobilized as well as their relationships and levels of integration with other governing institutions. A more detailed measure that captures different forms of military recruitment and organization, as well as a specific measure for military technology, could be used to examine how these affect enemy treatment.

While the quantitative analyses here did not show clear differences in proportional levels of violence, contrasting the literature on contemporary nation-state war with that on ancient states and non-state societies highlights the focus on extensive ideological justifications for extermination campaigns in the former. In their book on mass killing, Chirot and McCauley express this as a possible improvement, writing that ‘it might be seen as a sign of hope that, of all the reasons for massacring people, the first, simple convenience, is somewhat less acceptable today than in the past’ (Chirot & McCauley, 2010, p. 47). It is possible such changes would be quantifiable with more extensive data on norms of war, instances of mass violence and casualty numbers, and records of codified restraints. However, when this is not sufficient for the purposes of quantitative analysis, qualitative comparisons may be necessary to fully understand the dynamics behind a given behaviour, warfare-related or otherwise.

Although there are numerous theoretical reasons why there would be no clear relationships between social complexity and degree of indiscriminate violence, given these methodological challenges, the results of this set of analyses do not provide conclusive evidence against both hypotheses. However, one aim of this study was the construction of a scale that would capture cross-cultural variation in normal, socially acceptable levels of lethal violence towards enemies during war. Social complexity is a variable with a relatively consistent directional trend throughout human history. The extensive literature on social complexity and warfare tends not to focus on norms about how enemies in war are treated, but it provided a starting point from which to make predictions even if they were of unrealistically simple linear relationships. Collating available descriptions from the ethnographic and historical records was partly an exercise in determining the nature of these data and the extent to which they could be quantified as variables of interest, and can ideally be built on or inform other approaches.

## Supporting information

Basava supplementary material Basava supplementary material

## Supplementary material

The supplementary material for this article can be found at https://doi.org/10.1017/ehs.2025.10033

## Research transparency and reproducibility

The data and code used in this paper are available at https://github.com/kcbasava/historical-war-practices/tree/main/indiscriminate-killing. This includes the data set pre-imputation (inkill_preimputation.csv), with imputed data (inkill_imp25.csv), and with sources and evidence (inkill_dataevidence.xlsx).

## References

[ref1] Abou El Fadl, K. (2001). *Rebellion and violence in Islamic law*. Cambridge University Press.

[ref2] Arkush, E., & Tung, T. A. (2013). Patterns of war in the Andes from the Archaic to the Late Horizon: Insights from settlement patterns and cranial trauma. *Journal of Archaeological Research*, 21(4), 307–369. 10.1007/s10814-013-9065-1

[ref3] Bellah, R. N. (2011). *Religion in human evolution: From the paleolithic to the axial age*. Harvard University Press.

[ref4] Bellamy, A. J. (2012). Mass killing and the politics of legitimacy: Empire and the ideology of selective extermination. *Australian Journal of Politics & History*, 58(2), 159–180. 10.1111/j.1467-8497.2012.01630.x

[ref5] Brekke, T. (Ed.). (2005). *The ethics of war in Asian civilizations: A comparative perspective*. Routledge.

[ref6] Burch, E. S. (2005). *Alliance and conflict: The world system of the Iñupiaq Eskimos*. University of Calgary Press.

[ref7] Bürkner, P.-C. (2017). brms: An R package for Bayesian multilevel models using stan. *Journal of Statistical Software*, 80(1). 10.18637/jss.v080.i01

[ref8] Butler, S. M. (2018). Getting medieval on Steven Pinker. *Historical Reflections/Réflexions Historiques*, 44(1), 29–40. 10.3167/hrrh.2018.440105

[ref9] Chirot, D., & McCauley, C. (2010). *Why not kill them all? The logic and prevention of mass political murder* (New Edition). Princeton University Press.

[ref10] Cox, R. (2017). Expanding the history of the just war: The ethics of war in ancient Egypt. *International Studies Quarterly*, 61(2), 371–384. 10.1093/isq/sqx009

[ref11] Crevecoeur, I., Dias-Meirinho, M.-H., Zazzo, A., Antoine, D., & Bon, F. (2021). New insights on interpersonal violence in the Late Pleistocene based on the Nile valley cemetery of Jebel Sahaba. *Scientific Reports*, 11(1), 9991. 10.1038/s41598-021-89386-y 34045477 PMC8159958

[ref12] Crocker, W. H., & William, H. (1990). *The Canela (Eastern Timbira), I: an ethnographic introduction*. Smithsonian Institution Press. https://ehrafworldcultures.yale.edu/cultures/so08/documents/005

[ref13] Currie, T. E., & Mace, R. (2011). Mode and tempo in the evolution of socio-political organization: Reconciling “Darwinian” and “Spencerian” evolutionary approaches in anthropology. *Philosophical Transactions of the Royal Society B: Biological Sciences*, 366(1567), 1108–1117. 10.1098/rstb.2010.0318 PMC3049089 21357233

[ref14] Downes, A. B. (2006). Desperate times, desperate measures: The causes of civilian victimization in war. *International Security*, 30(4), 152–195. 10.1162/isec.2006.30.4.152

[ref15] Dwyer, P., & Micale, M. (2021). Chapter 1. Steven Pinker and the nature of violence in history. In P. Dwyer & M. Micale (Eds.), *The darker angels of our nature: Refuting the Pinker theory of history & violence* (pp. 1–22). Bloomsbury Academic. 10.5040/9781350148437

[ref16] Eck, K., & Hultman, L. (2007). One-sided violence against civilians in war: Insights from new fatality data. *Journal of Peace Research*, 44(2), 233–246. 10.1177/0022343307075124

[ref17] Ember, C., & Ember, M. (2013, December 3). Basic guide to cross-cultural research. *Human Relations Area Files – Cultural Information for Education and Research*. https://hraf.yale.edu/cross-cultural-research/basic-guide-to-cross-cultural-research/

[ref18] Ember, C. R., Adem, T. A., & Skoggard, I. (2013). Risk, uncertainty, and violence in Eastern Africa. *Human Nature*, 24(1), 33–58. 10.1007/s12110-012-9157-5 23242920

[ref19] Ember C R and Ember M. (1992). Warfare, Aggression, and Resource Problems: Cross-Cultural Codes. *Behavior Science Research*, 26(1–4), 169–226. 10.1177/106939719202600108

[ref20] Ember, C. R., & Ember, M. (2014). Chapter 1: Violence in the ethnographic record: Results of cross-cultural research on war and aggression. In D. L. Martin & D. W. Frayer (Eds.), *Troubled times: Violence and warfare in the past* (pp. 1–20). Gordon and Breach.

[ref21] Falk, D., & Hildebolt, C. (2017). Annual war deaths in small-scale versus state societies scale with population size rather than violence. *Current Anthropology*, 58(6), 805–813. 10.1086/694568

[ref22] Feinman, Gary. (2013) The emergence of social complexity. In *Cooperation and collective action: archaeological perspectives* (Ed.), Carballo David University Press of Colorado

[ref23] Ferguson, R. B. (2013). Pinker’s list: Exaggerating prehistoric war mortality. In D. P. Fry Ed., *War, peace, and human nature* (1st ed., pp. 112–131). Oxford University Press New York. 10.1093/acprof:oso/9780199858996.003.0007

[ref24] Ferguson, R. B., & Whitehead, N. L. (1992). Chapter 1: The violent edge of empire. In R. B. Ferguson (Ed.), *War in the tribal zone: Expanding states and indigenous warfare* (pp. 1–30). School of American Research Press. https://hdl.handle.net/2027/heb03246.0001.001

[ref25] Fibiger, L. (2018). The past as a foreign country. *Historical Reflections/Réflexions Historiques*, 44(1), 6–16. 10.3167/hrrh.2018.440103

[ref26] Fibiger, L., Ahlström, T., Meyer, C., & Smith, M. (2023). Conflict, violence, and warfare among early farmers in northwestern Europe. *Proceedings of the National Academy of Sciences*, 120(4), e2209481119. 10.1073/pnas.2209481119 PMC9942812 36649427

[ref27] Fischer, J. L. (1958). *Native land tenure in the Truk District* (pp. 161–215). Office of the High Commisioner, Trust Territory of the Pacific Islands. https://ehrafworldcultures.yale.edu/cultures/or19/documents/036

[ref28] Fischer, M. D., & Ember, C. R. (2018). Big data and research opportunities using HRAF databases. In S.-H. Chen (Ed.), *Big data in computational social science and humanities* (pp. 323–336). Springer International Publishing. 10.1007/978-3-319-95465-3_17

[ref29] Fry, D. P. (2013). War, Peace, and Human Nature: The Convergence of Evolutionary and Cultural Views.Oxford University Press.

[ref30] Glowacki, L. (2024). The evolution of peace. *Behavioral and Brain Sciences*, 47, e1. 10.1017/S0140525X22002862 36524358

[ref31] Glowacki, L., Wilson, M. L., & Wrangham, R. W. (2020). The evolutionary anthropology of war. *Journal of Economic Behavior & Organization*, 178, 963–982. 10.1016/j.jebo.2017.09.014

[ref32] Gronenborn, D. (2006). Climate change and socio-political crises: Some cases from Neolithic central Europe. *Journal of Conflict Archaeology*, 2(1), 13–32. 10.1163/157407706778942231

[ref33] Haas, J. (2001). Warfare and the evolution of culture. In G. M. Feinman & T. D. Price (Eds.), *Archaeology at the Millennium* (pp. 329–350). Springer US. 10.1007/978-0-387-72611-3_9

[ref34] Hames, R. (2019). Pacifying hunter-gatherers. *Human Nature*, 30(2), 155–175. 10.1007/s12110-019-09340-w 30953274

[ref35] Harff, B. (2003). No lessons learned from the holocaust? Assessing risks of genocide and political mass murder since 1955. *American Political Science Review*, 97(01), 57–73. https://www.cambridge.org/core/journals/american-political-science-review/article/no-lessons-learned-from-the-holocaust-assessing-risks-of-genocide-and-political-mass-murder-since-1955/FBA37FA563AC35E1CB6F7B57F8140F2C

[ref36] Hasluck, M. M. H., Hutton, J. H., & John, H. (1954). *The unwritten law in Albania*. University Press. https://ehrafworldcultures.yale.edu/document?id=eg01-010

[ref37] Hatch, M. A. (2017). Politics and social substitution in total war: Exploring the treatment of combatants and noncombatants during the Mississippian period of the Central Illinois Valley. In D. L. Martin & C. Tegtmeyer (Eds.), *Bioarchaeology of women and children in times of war* (pp. 49–69). Springer International Publishing. 10.1007/978-3-319-48396-2_4

[ref38] Hobhouse, L. T., Wheeler, G. C., & Ginsberg, M. (1915). The material culture and social institutions of the simpler peoples: An essay in correlation. *The Sociological Review*, a7(4), 332–368. 10.1111/j.1467-954X.1914.tb02396.x

[ref39] Johnson, J. T. (1981). *Just war tradition and the restraint of war: A moral and historical inquiry*. Princeton University Press.

[ref40] Jones, D. M. (1970). *A study of social and economic problems in Unalaska, an Aleut village*. University Microfilms. https://ehrafworldcultures.yale.edu/document?id=na06-070

[ref41] Karsten, R. (1935). *The head-hunters of Western Amazonas: the life and culture of the Jibaro Indians of eastern Ecuador and Peru*. Helsingfors. https://ehrafworldcultures.yale.edu/document?id=sd09-001

[ref42] Keeley, L. H. (1996). *War before civilization: The myth of the peaceful savage*. Oxford University Press USA. http://ebookcentral.proquest.com/lib/oxford/detail.action?docID=694019

[ref43] Kelly, R. C. (2000). *Warless societies and the origin of war*. University of Michigan Press.

[ref44] Kiernan, B. (2007). *Blood and soil: A world history of genocide and extermination from Sparta to Darfur*. Yale University Press.

[ref45] Kim, N. C. (2012). Angels, illusions, hydras, and chimeras: Violence and humanity. *Reviews in Anthropology*, 41(4), 239–272. 10.1080/00938157.2012.732511

[ref46] Kim, N. C., & Kissel, M. (2018). *Emergent warfare in our evolutionary past*. Routledge, Taylor & Francis Group.

[ref47] Kim, N. C., Kusimba, C. M., & Keeley, L. H. (2015). Coercion and warfare in the rise of state societies in southern Zambezia. *African Archaeological Review*, 32(1), 1–34. 10.1007/s10437-015-9183-x

[ref48] Kirby K R et al. (2016). D-PLACE: A Global Database of Cultural, Linguistic and Environmental Diversity. PLoS ONE, 11(7), e0158391 10.1371/journal.pone.0158391, https://doi.org/10.1371/journal.pone.0158391.g001, https://doi.org/10.1371/journal.pone.0158391.g002, https://doi.org/10.1371/journal.pone.0158391.t001, https://doi.org/10.1371/journal.pone.0158391.s001, https://doi.org/10.1371/journal.pone.0158391.s002, https://doi.org/10.1371/journal.pone.0158391.s003 27391016 PMC4938595

[ref49] Kissel, M., & Kim, N. C. (2019). The emergence of human warfare: Current perspectives. *American Journal of Physical Anthropology*, 168(S67), 141–163. 10.1002/ajpa.23751 30575025

[ref50] Kurz, A. S. (2019). *Statistical rethinking with brms, ggplot2, and the tidyverse: Version 1.0.1*. https://bookdown.org/ajkurz/Statistical_Rethinking_recoded/

[ref51] LeBlanc, S. A., & Register, K. E. (2003). *Constant battles: Why we fight* (1st edition). St. Martins’ Griffin.

[ref52] Mann, M. (2018). Have wars and violence declined? *Theory and Society*, 47(1), 37–60. 10.1007/s11186-018-9305-y

[ref53] McElreath, R. (2020). *Statistical rethinking: A Bayesian course with examples in R and stan* (2nd ed.). Taylor and Francis, CRC Press.

[ref54] Meyer, C., Kürbis, O., Dresely, V., & Alt, K. W. (2018). Patterns of collective violence in the early Neolithic of central Europe. In A. Dolfini, R. J. Crellin, C. Horn & M. Uckelmann (Eds.), *Prehistoric warfare and violence: Quantitative and qualitative approaches* (pp. 21–38). Springer International Publishing. 10.1007/978-3-319-78828-9_2

[ref55] Mirazón Lahr, M., Rivera, F., Power, R. K., Mounier, A., Copsey, B., Crivellaro, F., Edung, J. E., Fernandez, J. M. M., Kiarie, C., Lawrence, J., Leakey, A., Mbua, E., Miller, H., Muigai, A., Mukhongo, D. M., Van Baelen, A., Wood, R., Schwenninger, J.-L., Grün, R., & Foley, R. A. (2016). Inter-group violence among early Holocene hunter-gatherers of West Turkana, Kenya. *Nature*, 529(7586), 394–398. 10.1038/nature16477 26791728

[ref56] Murdock G. P., & White D. R. (2025). *D-PLACE dataset derived from Murdock and White (1969) Standard Cross-Cultural Sample* [Data set]. D-PLACE. https://zenodo.org/records/17601873

[ref57] Murdock, G. P., Textor, R., Barry III, H. White, D. R., Gray, J. P., Divale, W. T. (2025). *D-PLACE dataset derived from Murdock et al. (1999) Ethnographic Atlas [Data set]*. D-PLACE. https://zenodo.org/records/17602181

[ref58] Naroll, R. (1967). The proposed HRAF probability sample. *Behavior Science Notes*, 2(2), 70–80. 10.1177/106939716700200202

[ref59] Nyseth Brehm, H. (2016). State context and exclusionary ideologies. *American Behavioral Scientist*, 60(2), 131–149. 10.1177/0002764215607579

[ref60] Oka, R. C., Kissel, M., Golitko, M., Sheridan, S. G., Kim, N. C., & Fuentes, A. (2017). Population is the main driver of war group size and conflict casualties. *Proceedings of the National Academy of Sciences*, 114(52). 10.1073/pnas.1713972114 PMC5748198 29229847

[ref61] Otterbein, K. F. (1970). *The evolution of war: A cross-cultural study*. HRAF Press.

[ref62] Otterbein, K. F. (1999). A history of research on warfare in anthropology. *American Anthropologist*, 101(4), 794–805. 10.1525/aa.1999.101.4.794

[ref63] Otterbein, K. F. (2000). Killing of captured enemies: A cross‐cultural study. *Current Anthropology*, 41(3), 439–443. 10.1086/300150 10768886

[ref64] Pinker, S. (2011). *The better angels of our nature: Why violence has declined*. Penguin Books.

[ref65] Pospisil, L. J. (1958). *Kapauku Papuans and their law*. New Haven: Human Relations Area Files Press.

[ref66] *Prior Choice Recommendations*. (n.d.). GitHub. Retrieved August 6, 2025, from https://github.com/stan-dev/stan/wiki/Prior-Choice-Recommendations

[ref67] Raymond, Gregory A. (2010). The Greco-Roman Roots of the Western Just War Tradition. In H. M. Hensel (Ed.), *The Prism of Just War: Asian and Western Perspectives on the Legitimate Use of Military Force* (pp. 7–27). London: Routledge

[ref68] Singer, P. (2011). *The expanding circle: Ethics, evolution, and moral progress* (1st Princeton University Press pbk. edition). Princeton University Press.

[ref69] Smith, R. S. (1989). *Warfare & diplomacy in pre-colonial West Africa* (2nd edition). University of Wisconsin Press.

[ref70] Snyder, T. J., & Arkush, E. (2024). Political organization and gender predict violence in the Andean archaeological record. *Proceedings of the National Academy of Sciences*, 121(44), e2410078121. 10.1073/pnas.2410078121 PMC11536129 39432790

[ref71] Solometo, J. (2008). Conflict and culture change in central Arizona. In E. Arkush & M. W. Allen (Eds.), *The archaeology of warfare: Prehistories of raiding and conquest* (pp. 23–65). University Press of Florida.

[ref72] Straight, B. (2017). Uniquely human: Cultural norms and private acts of mercy in the war zone. *American Anthropologist*, 119(3), 491–505. 10.1111/aman.12905

[ref73] Straus, S. (2012). “Destroy them to save us”: Theories of genocide and the logics of political violence. *Terrorism and Political Violence*, 24(4), 544–560. 10.1080/09546553.2012.700611

[ref74] Syed, M. (2013). Jihad in classical Islamic legal and moral thought. In J. Neusner, B. Chilton & R. E. Tully (Eds.), *Just war in religion and politics* (pp. 135–162). University Press of America.

[ref75] Textor, J., van der Zander, B., Gilthorpe, M. S., Liśkiewicz, M., & Ellison, G. T. H. (2017). Robust causal inference using directed acyclic graphs: The R package ‘dagitty’. *International Journal of Epidemiology*, dyw341. 10.1093/ije/dyw341 28089956

[ref76] Turchin, P., Brennan, R., Currie, T. E., Feeney, K., François, P., Hoyer, D., & Peregrine, P. (2015). Seshat: The global history databank. *Cliodynamics: The Journal of Quantitative History and Cultural Evolution*. 10.21237/C7clio6127917

[ref77] Turchin, P., Currie, T. E., Whitehouse, H., Francois, P., Feeney, K., Mullins, D., Hoyer, D., Collins, C., Grohmann, S., Savage, P., Mendel-Gleason, G., Turner, E., Dupeyron, A., Cioni, E., Reddish, J., Levine, J., Jordan, G., Brandl, E., Williams, A., & Spencer, C. (2018). Quantitative historical analysis uncovers a single dimension of complexity that structures global variation in human social organization. *Proceedings of the National Academy of Sciences of the United States of America*, 115(2), E144–E151. 10.1073/pnas.1708800115 29269395 PMC5777031

[ref78] Underhill, A. P. (2008). Warfare and the development of states in China. In E. N. Arkush & M. W. Allen (Eds.), *The archaeology of warfare: Prehistories of raiding and conquest* (pp. 253–285). University Press of Florida.

[ref79] Valentino, B. A. (2014). Why we kill: The political science of political violence against civilians. *Annual Review of Political Science*, 17(1), 89–103. 10.1146/annurev-polisci-082112-141937

[ref80] van Buuren, S., & Groothuis-Oudshoorn, K. (2011). mice: Multivariate imputation by chained equations in R. *Journal of Statistical Software*, 45(3), 1–67. 10.18637/jss.v045.i03

[ref81] Wiessner, P. (2019). Collective action for war and for peace a case study among the Enga of Papua New Guinea. *Current Anthropology*, 60(2), 224–244. 10.1086/702414

[ref82] Zefferman, M. R., & Mathew, S. (2020). An evolutionary theory of moral injury with insight from Turkana warriors. *Evolution and Human Behavior*, 41(5), 341–353. 10.1016/j.evolhumbehav.2020.07.003

