# Supplementary material for: Social complexity is not strongly predictive of indiscriminate killing of wartime enemies in a cross-cultural sample
Source: Evol Hum Sci. 2025 Dec 22;8:e4. doi: 10.1017/ehs.2025.10033 (PMC12936596; doi:10.1017/ehs.2025.10033)
Supplement: Basava supplementary material [file S2513843X25100339sup001.docx]

**Supplementary Material for ‘Social complexity is not strongly predictive of indiscriminate killing of wartime enemies’**

*Variable descriptions:*

Variable: Population size

Dataset variable name: pop_res1

Description: Population estimates for each society at the date range being coded, binned according to the number of places.

Original codes: (0-99=1; 100-999=2; 1000-9999=3; 10000-99999=4; 100000-999999=5; 1000000-9999999=6;10000000-99999999=7; 100000000-999999999=8)

Codes for analyses: 1-8, standardized (mean centered at 0, sd = 1)

Variable: Political expansion

Dataset variable name: ter_res

Description: Whether warfare conducted by the society increased the territory or population under its political control. It indicates deliberate attempts to expand political control but not decentralized expansion into land e.g. for hunting, grazing, or farming that involves fighting.

Original codes: yes, no, unclear

Codes for analyses: 0/1

Variable: Degree of political centralization

Dataset variable name: pol_cent

Description: How centralized is the cultural unit to which the practices coded for the most external level of war refer.

Original codes: This variable was coded based on EA and Seshat codes.

1. acephalous (absence of any political organization; equivalent to EA090); 2. autonomous communities (local political integration transcending kin groups, includes societies coded under EA090 autonomous local communities and peace groups); 3. loose/nominal/minimal states (a central government with some control or authority over relatively autonomous regional rulers, equivalent to EA090 minimal states and Seshat nominal, loose polities); 4. confederated/feudal states (the central government has more control over regional rulers but not complete authority); 5. unitary states (as defined by the Seshat variable- regional governors are appointed and removed by the central authorities, taxes are imposed by, and transmitted to the center)

Codes for analyses: 1-5

Variable: Hierarchical governing levels

Dataset variable name: ext_hier

Description: Number of administrative/governing levels beyond the local community; following the Ethnographic Atlas variable 33 ‘Jurisdictional hierarchy beyond local community’ generally used as a measure of political complexity in cross-cultural studies.

Original codes: Coded from 0 to 5 rather than the original 1 to 5 in EA033 to make code equal to the number of levels and to accommodate larger polities in the dataset. From description on D-place: 0 (=EA033 1, acephalous, no political authority beyond community, autonomous bands and villages); 1 (one level, petty chiefdoms); 2 (two levels, larger chiefdoms); 3 (three levels, states); 4 (four levels, large states); 5 (my addition, five or more levels, empires)

Codes for analyses: 0-5, standardized (mean centered at 0, sd = 1)

Variable: Social complexity

Dataset variable name: PC1

Description: Overall measure of sociopolitical complexity created from population size, hierarchical governing levels, and political centralization variables.

Original codes: NA

Codes for analyses: first component from PCA of above variables, standardized (mean centered at 0, sd = 1)

Variable: Presence of formal military organization

Dataset variable name: mil_res

Description: Whether there is a formal standing army or military organization, or whether warriors are mobilized through social relations such as friends or kin.

Original codes: 0/1

Codes for analyses: 0/1

Variable: Enemies killed in war

Dataset variable name: kill_ext

Description: Whether enemy individuals in the following age/gender categories tend to be killed during war: male or female infants/toddlers; children; younger adults; older adults

Original codes: 1-8. 1 point is added for each age/gender category, with the maximum being 8 (everyone). Ranges are used for some societies when there is ambiguity or overlap across categories.

Codes for analyses: 1-8

Variable: Enemies killed measurement error

Dataset variable name: kill_se

Description: The difference between the mean and maximum/minimum of range of enemies killed for each society.

Original codes: NA

Codes for analyses: 0-3

Other descriptive variables (not used in analyses):

Variable: time_period

Description: The time period to which the other variables coded refer to, based on the date ranges covered by the sources referenced. For historical societies this generally is the start and end dates of a particular political organization, and may therefore be approximate. For ethnographically studied societies it is generally taken from the coverage dates given in eHRAF for the ethnographies used to code the other variables.

Codes: Year range

Variable: timeperiod_source

Description: The source for the start and end dates recorded. 'source' means that these were taken from the same sources used to code the indiscriminate killing ('killed_ext') variable. In other cases, another source was recorded.

Variable: pop_source

Description: Sources for population estimates. ‘eHRAF’ indicates the society’s cultural summary page; ‘EA202’ indicates variable 202 population size in the Ethnographic Atlas; ‘Seshat’ indicates the polity population variable on the society’s Seshat page; other sources cited specifically

*Regional autocorrelation*


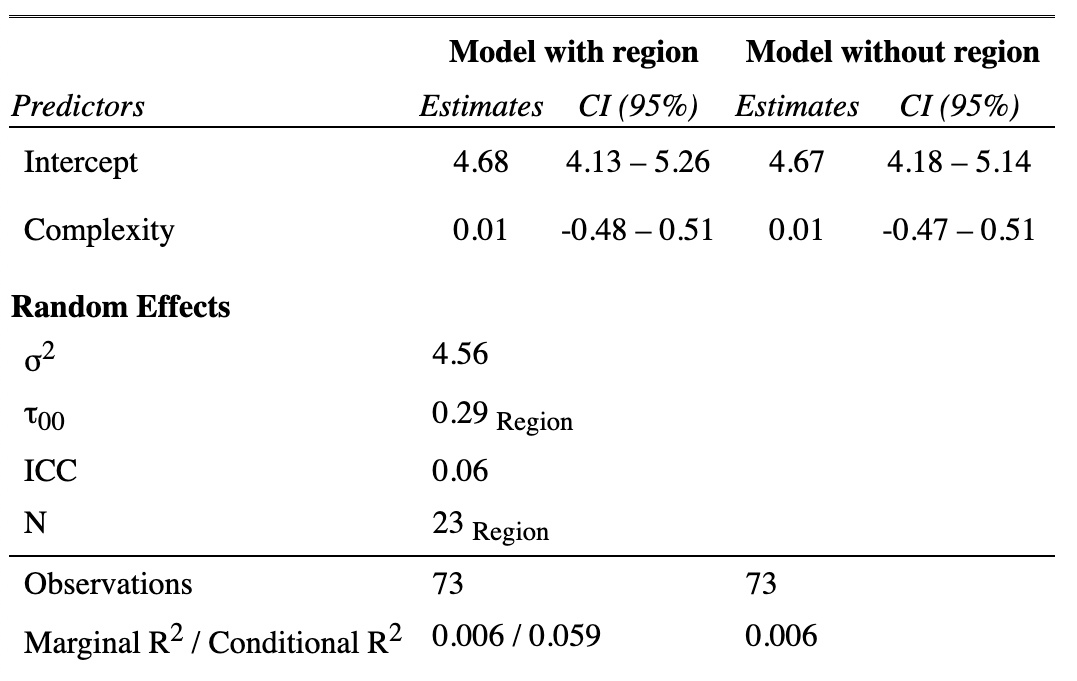


*Table S1. Comparison of simplified model, not including error term, incorporating region as a categorical varying intercept (first column) with simplified model without region (second column).*

*Temporal autocorrelation*


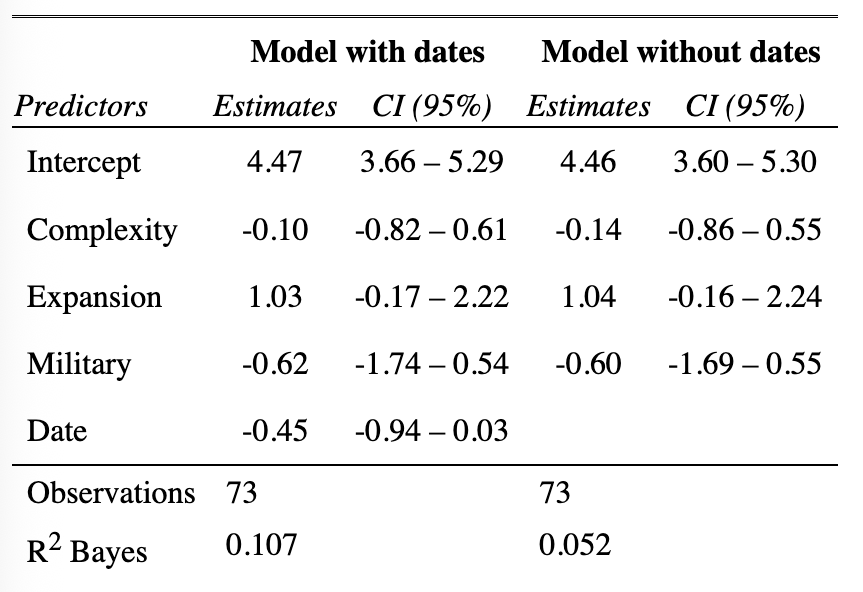


*Table S2. Estimates from model with mean standardized date added as predictor (first column), compared with model without date as a predictor (column 2).*

Keywords for searching eHRAF: enem* kill* death die demolish* violen* massacr* slaughter* exterminat* annihilat* genocid* dispatch* tortur* destroy* suffer* cruel* victim* harm injur* atrocit* violat* brutal* feroc* age sex old young gender girl* boy* women child children infant infants surrender* protect* defeat* convention* limit* norm norms normative unarmed innocent lenien* pity compassion* charit* quarter sympathy immun* mercy merciful spare* discriminate* indiscriminate taboo sanctity sanctuary asylum conduct civilian* noncombatant* refuge inviolab* restrain* clemency refrain* ethic* moral*
